# Multilevel Intervention and Human Papillomavirus Vaccination Disparities: A Secondary Analysis of a Cluster Randomized Trial

**DOI:** 10.1001/jamanetworkopen.2025.18895

**Published:** 2025-07-07

**Authors:** Wei Yi Kong, Lila J. Finney Rutten, Jeph Herrin, Jennifer L. St Sauver, Gregory D. Jenkins, Joan M. Griffin, Robert M. Jacobson

**Affiliations:** 1 Division of Epidemiology, Mayo Clinic, Rochester, Minnesota; 2 Division of Community Internal Medicine, Geriatrics, and Palliative Care, Mayo Clinic, Rochester, Minnesota; 3 Section of Cardiovascular Medicine, Yale University School of Medicine, New Haven, Connecticut; 4 Flying Buttress Associates, Charlottesville, Virginia; 5 Division of Biomedical Statistics and Informatics, Mayo Clinic, Rochester, Minnesota; 6 Division of Health Care Delivery Research and Robert D. and Patricia E. Kern Center for Science of Health Care Delivery, Mayo Clinic, Rochester, Minnesota; 7 Division of Community Pediatric and Adolescent Medicine, Mayo Clinic, Rochester, Minnesota; 8 Division of Pediatric Infectious Diseases, Mayo Clinic, Rochester, Minnesota

## Abstract

**Importance:**

Uptake of human papillomavirus (HPV) vaccination varies by characteristics, exposing some children to higher HPV cancer risks than others.

**Objective:**

To examine whether the effectiveness of a multilevel intervention on HPV vaccination differed by race and ethnicity, rurality, and Area Deprivation Index (ADI) in children ages 11 to 12 years.

**Design, Setting, and Participants:**

A stepped-wedge cluster randomized trial was conducted from April 2018 to August 2022 among children at 6 Mayo Clinic primary care practices in Minnesota to improve HPV vaccination. This secondary analysis was performed from March to June 2024.

**Intervention:**

A multilevel intervention that included parent reminder/recall letters, which alerted parents of children due or past due for vaccination, and health care professional audit/feedback reports, which alerted health care professionals of their own vaccination rates.

**Main Outcome and Measure:**

Vaccine initiation (first dose of the 2-dose HPV vaccine) and vaccine completion (second dose) were the primary study outcomes. In this secondary analysis, the effect of the intervention on HPV vaccine initiation and completion by race and ethnicity, rurality, and ADI quartiles (Qs) was assessed.

**Results:**

A total of 6232 children aged 11 to 12 years (3285 [52.7%] male; 3481 [55.9%] aged 11 years and 2751 [44.1%] aged 12 years) were included in the analysis. Of the study participants, 304 (4.9%) were Asian, 561 (9.0%) Black, 146 (2.3%) Hispanic, 4501 (72.2%) White, and 720 (11.6%) other, including American Indian or Alaskan Native, Native Hawaiian or Pacific Islander, Other Pacific Islander, Samoan, unable to provide, unknown, chose not to disclose, or other unspecified. A total of 5434 participants (87.2%) were urban residents, and 2794 (44.8%) resided in ADI Q2 areas. With usual care, HPV vaccine initiation and completion rates were significantly lower with each increasing ADI quartile (initiation: Cochran-Armitage test for trend [SE], −0.02 [0.01]; *P* < .001; completion: Cochran-Armitage test for trend [SE], −0.05 [0.01]; *P* < .001) but did not differ by children’s race and ethnicity or rurality. With the intervention, vaccine initiation increased significantly for most children (range of rates, 9.2% [95% CI, 5.2%-13.3%] to 24.0% [95% CI, 7.5%-40.6%]) except those with Black race, in rural settings, and in ADI Q4 (highest area deprivation); vaccine completion increased significantly for most children (range of rates, 19.4% [95% CI, 5.5%-33.3%] to 31.2% [95% CI, 12.1%-50.3%]) except for those in ADI Q4.

**Conclusions and Relevance:**

In this secondary analysis of a cluster randomized trial, a multilevel intervention was associated with increased HPV vaccination for most children but had limited effect for those residing in areas of highest deprivation. Future research should explore other intervention strategies that would effectively promote HPV vaccination among families in socioeconomically disadvantaged areas to reduce HPV vaccination disparities.

**Trial Registration:**

ClinicalTrials.gov Identifier: NCT03501992

## Key Points

**Question:**

Does the effect of a multilevel intervention to improve human papillomavirus (HPV) vaccine uptake among children differ by race and ethnicity, rurality, and area deprivation?

**Findings:**

In this secondary analysis of a cluster randomized trial of 6232 children, a combination of parent reminder/recall letters and health care professional audit/feedback was associated with improved HPV vaccine uptake among most children aged 11 to 12 years but had limited impact among those residing in the most socioeconomically disadvantaged areas.

**Meaning:**

More efforts are needed to develop and test additional or alternative intervention strategies to increase HPV vaccine initiation and completion among children residing in the most socioeconomically disadvantaged areas.

## Teaser

This secondary analysis of a cluster randomized trial examines the effect of parent reminder/recall letters and clinician audit/feedback reports on pediatric human papillomavirus vaccination by race and ethnicity, rurality, and area deprivation.

## Introduction

Infections with high-risk human papillomavirus (HPV) contributed to approximately 37 000 new cancer cases in the US every year between 2016 and 2020.^1^ Routine HPV vaccination is recommended at 11 to 12 years of age to lower the risks of HPV-attributable cancers in children.^2^ Despite the wide availability and robust safety evidence of the vaccine,^3^ current up-to-date HPV vaccine coverage at 50% is below the national goal of achieving 80% coverage.^4,5^ Furthermore, not all children have the same level of vaccine coverage, with HPV vaccine initiation and completion differing by children’s race and ethnicity, rurality, and area deprivation.^6,7,8,9,10,11,12^ For example, most studies^7,8,9,10,11^ noted that children of races other than White are more likely to initiate HPV vaccination but less likely to complete vaccination than their White counterparts. Lower vaccine initiation is observed with rural residence and higher area deprivation, whereas vaccine completion has mixed associations with rurality but not associated with area deprivation.^8,9,10,12^ These disparities in vaccine coverage disproportionately place some children at higher HPV cancer risks than others.

The Community Preventive Services Task Force recommends interventions—such as reminder/recall notifications, which alert patients or parents of children due or past due for vaccination, and audit/feedback notifications, which alert health care professionals of their own vaccination rates—to promote vaccination.^13,14,15,16^ Clinical trial evidence supports the effectiveness of parent reminder/recalls and health care professional audit/feedback in increasing adolescent vaccination rates.^17,18,19,20,21^ Our clinical trial similarly found evidence for reminder/recalls and audit/feedback in improving HPV vaccination, wherein both interventions doubled the odds of vaccine uptake compared with usual care and are more effective than each intervention alone.^22^ Although our trial supports implementing these interventions in combination, whether the interventions had a differential impact on HPV vaccine uptake among children is unclear. In light of the known HPV vaccination disparities, we hypothesized that the parent reminder/recall and health care professional audit/feedback interventions reduced vaccination disparities across children.

## Methods

We conducted a post hoc secondary analysis of a cluster randomized trial that was conducted from April 2018 to August 2022 among 6 Mayo Clinic primary care practices in southeast Minnesota.^22^ The Mayo Clinic institutional review board approved the trial and this secondary analysis. The Mayo Clinic Institutional Review Board deemed the original trial to be minimal risk and waived informed consent. The trial followed the Consolidated Standards of Reporting Trials (CONSORT) reporting guideline.

Briefly, the trial featured a stepped-wedge factorial design to examine the impact of parent reminder/recall letters and health care professional audit/feedback reports, separately and in combination, on adolescent HPV vaccination. The content and delivery of reminder/recall letters and audit/feedback reports were informed by previous work and thereafter refined based on our interviews and focus groups with parents and health care professionals.^23^ The 6 participating practices were assigned to the interventions using stratified randomization based on patient volume. Each practice was exposed to the intervention components individually and in combination during 4 trial steps (eFigure in Supplement 1). Only our statisticians (J.H. and G.D.J.) were aware of the practice allocation sequence until the interventions were assigned. Patients and health care professionals were blinded to the randomization. Each step of the trial was 12 months long, starting with usual care from April 2018 to March 2019. At each trial step, children empaneled to practices assigned to the reminder/recall intervention were assessed monthly for HPV vaccination eligibility; a reminder/recall letter was mailed every month during the trial step to parents of children who became eligible for a vaccine dose. Each parent received a 1-time mailing of the letter. For practices assigned to the audit/feedback intervention at each trial step, health care professionals were sent a monthly report via the internal campus mailing system detailing the 3-month mean HPV vaccination rates achieved by the individual health care professional and their practice.

The study was paused from April to August 2020 due to the COVID-19 pandemic; hence, the final step when all practices were exposed to both interventions was from September 2021 to August 2022. Eligible patients were those empaneled to 1 of the 6 practices who were aged 11 to 12 years and due for at least 1 dose to initiate and/or complete HPV vaccination based on their electronic health record (EHR). To evaluate our interventions, we extracted eligible adolescent patients’ HPV vaccination status and demographic and residential information from their EHR.

### Measures

We focused on demographic (race and ethnicity) and geographic (rurality and area deprivation) characteristics that were documented to differ in HPV vaccine uptake.^6,7,8,9,10,11,12^ We obtained parents’ self-reported information on their children’s race and ethnicity from the EHR as part of the original trial. Extracted race and ethnicity data were in categories of Asian, Black, Hispanic, White, and other (including American Indian and Alaskan Native, Native Hawaiian and Pacific Islander, Other Pacific Islander, Samoan, unable to provide, unknown, chose not to disclose, and other unspecified). We also extracted patient addresses from the EHR to classify each adolescent by rurality using the 2010 rural-urban commuting area codes based on US Census tracts.^24^ Children in US Census tracts labeled as rural-urban commuting area codes 1 and 2 were classified as urban residents and those located in US Census tracts in codes 3 to 10 were defined as rural residents. We also used patient addresses to assign Area Deprivation Index (ADI) using the 2021 version 4.01.1 ADI from the Neighborhood Atlas.^25,26^ ADI is an area-level composite that characterizes socioeconomic status and is derived from 17 US Census variables (eg, family income and unemployment rate).^27^ Using US Census mapping data from the American Community Survey 2017-2021,^28^ ADI is scaled at the US Census block level from 1 to 100 national percentile, with higher percentiles indicating higher area deprivation. Using this national percentile scale, we recategorized assigned ADI into quartiles (Qs) 1 to 4 that represent lowest (Q1) to highest (Q4) area deprivation based on national distribution to meaningfully assess the differential impact of the intervention by ADI.

### Outcomes

We assessed HPV vaccine initiation (first of 2 doses) and completion (second dose). Patients were assessed for eligibility to initiate or complete vaccination during their 11th or 12th birthday month. We determined a positive vaccination outcome as having received a vaccine dose (first dose for initiation and second dose for completion) during the remaining months of the trial step. We examined vaccine initiation and completion separately, given the different patterns in vaccination outcomes previously observed.^6,7,8,9,10,11,12^ We calculated HPV initiation and completion rates by estimating the probability of change in the vaccination outcomes for each race and ethnicity, rurality, and area deprivation characteristic.

### Statistical Analysis

As previously reported,^22^ our trial observed the highest increase in HPV vaccination over usual care with the implementation of both parent reminder/recall and health care professional audit/feedback. Therefore, for this analysis, we constrained our study sample to patients included in either usual care or combined intervention study periods (eTable 1 in Supplement 1). We included in our outcome analysis all patients who were empaneled to a participating practice at the time of their 11th or 12th birthday month. Because cluster randomization can produce systematic differences in individual-level characteristics, we calculated standardized mean differences (SMDs) between patients in the usual care and intervention groups, using SMD greater than 0.2 as a threshold for including characteristics in our final models.^29,30,31^ To assess demographic and geographic differences in HPV vaccination rates at usual care, we calculated vaccine initiation and completion rates for each stratum of race and ethnicity, rurality, and ADI quartile and reported odds ratios (ORs) with 95% CIs. We assessed trends in vaccine initiation and completion across ADI quartiles with Cochran-Armitage tests for trend.

To examine the intervention effect on vaccine initiation and completion, we estimated mixed-effects logistic regression models with the intervention as the fixed effect and the practice as the random effect, stratified by study participant characteristics. In these models, we did not include trial step as a fixed or random effect given the full collinearity with the intervention periods. We reported the marginal rates, ORs (95% CIs), and *P* values for each comparison as well as the intracluster correlation for each model. Because of the small number of practices, estimation of between-practice variance may give biased results.^32^ Therefore, we conducted sensitivity analyses that replicated all models using fixed-effects models. Fixed-effects models account conservatively for clustering of outcomes and are appropriate for stepped-wedge cluster randomized trials^33^ and are least biased and more efficient than other models when there are small clusters and no cluster level predictors.^34^

To test for significant differences in the intervention effect on vaccine initiation and completion across children, we estimated 3 mixed-effects models, 1 each for race and ethnicity, rurality, and ADI quartile, including all patients, with interaction terms between the intervention and the participant characteristic. We specified non-Hispanic White, urban, and lowest ADI quartile (Q1) as the respective reference groups for race and ethnicity, rurality, and ADI. We used each model to estimate the marginal rates for each interaction group.

We performed all statistical tests for this study from March to June 2024 using Stata software, version 18 (StataCorp) and determined statistical significance with a 2-tailed *P* < .05. Our analysis did not include data from April to August 2020 when the trial was paused due to the COVID-19 pandemic.

## Results

We included 6232 children aged 11 to 12 years (3481 [55.9%] aged 11 years and 2751 [44.1%] aged 12 years) from the 6 practices in the analysis. The number of children empaneled to each practice is shown in the eFigure in Supplement 1. None of the practices dropped out during the trial. Most children were eligible to initiate HPV vaccination (4443 [71.3%]) and were male (3285 [52.7%] vs 2947 [47.3%] female) and urban residing (5434 [87.2%]). Regarding race and ethnicity, 304 (4.9%) were Asian, 561 (9.0%) Black, 146 (2.3%) Hispanic, 4501 (72.2%) White, and 720 (11.6%) other. Most children resided in ADI Q2 (2794 [44.8%]), with few residing in the highest-deprivation areas (ADI Q4: 264 [4.2%]). Children in the usual care (n = 3752) and combined intervention (n = 2660) study periods differed by age, sex, rurality, and ADI (Table 1). With usual care, HPV vaccine initiation and completion rates were significantly lower with each increasing ADI quartile (initiation: Cochran-Armitage test for trend [SE], −0.02 [0.01]; *P* < .001; completion: Cochran-Armitage test for trend [SE], −0.05 [0.01]; *P* < .001) but did not differ by children’s race and ethnicity or rurality (Table 2).

**Table 1.** Characteristics of the Study Participants

Characteristic	No. (%) of participants			SMD
	Overall (N = 6232)	Usual care (n = 3572)	Intervention (n = 2660)	
HPV vaccine eligibility				
Initiation	4443 (71.3)	2738 (76.7)	1705 (64.1)	0.28
Completion	1789 (28.7)	834 (23.3)	955 (35.9)	
Age, y				
11	3481 (55.9)	1984 (55.5)	1497 (56.3)	0.02
12	2751 (44.1)	1588 (45.5)	1163 (43.7)	
Sex				
Male	3285 (52.7)	1905 (53.3)	1380 (51.9)	0.03
Female	2947 (47.3)	1667 (46.7)	1280 (48.1)	
Race and ethnicity				
Asian	304 (4.9)	197 (5.5)	107 (4.0)	0.13
Black	561 (9.0)	329 (9.2)	232 (8.7)	
Hispanic	146 (2.3)	71 (2.0)	75 (2.8)	
White	4501 (72.2)	2517 (70.5)	1984 (74.6)	
Other^a^	720 (11.6)	458 (12.8)	262 (9.8)	
Rurality^b^				
Urban	5434 (87.2)	3113 (87.2)	2321 (87.3)	0.02
Rural	438 (7.0)	260 (7.3)	178 (6.7)	
Area Deprivation Index^c^				
Q1	1090 (17.5)	641 (17.9)	449 (16.9)	0.05
Q2	2794 (44.8)	1597 (44.7)	1197 (45.0)	
Q3	1712 (27.5)	967 (27.1)	745 (28.0)	
Q4	264 (4.2)	161 (4.5)	103 (3.9)	

Abbreviations: HPV, human papillomavirus; Q, quartile (Q4 represents highest area deprivation); SMD, standardized mean difference.

^a^ Includes American Indian and Alaskan Native, Native Hawaiian and Pacific Islander, Other Pacific Islander, Samoan, unable to provide, unknown, chose not to disclose, and other unspecified.

^b^ Data missing for 360 participants.

^c^ Data missing for 372 participants.

**Table 2.** Human Papillomavirus Vaccine Initiation and Completion Rates at Usual Care by Characteristics of the 3572 Study Participants

Characteristic	Vaccine initiation		Vaccine completion	
	Rate, % (No./total No.)	OR (95% CI)	Rate, % (No./total No.)	OR (95% CI)
Race and ethnicity				
Asian	15.9 (22/138)	0.84 (0.50-1.35)	28.8 (17/59)	0.61 (0.32-1.14)
Black	13.8 (34/246)	0.71 (0.47-1.04)	30.1 (25/83)	0.65 (0.38-1.10)
Hispanic	13.5 (7/52)	0.69 (0.26-1.55)	36.8 (7/19)	0.89 (0.29-2.49)
White	18.5 (366/1978)	1.0 [Reference]	39.7 (214/539)	1.0 [Reference]
Other^a^	16.4 (53/324)	0.86 (0.62-1.19)	27.6 (37/134)	0.58 (0.37-0.89)
Rurality				
Urban	17.9 (427/2387)	1.0 [Reference]	35.7 (259/726)	1.0 [Reference]
Rural	18.3 (36/197)	1.03 (0.68-1.51)	42.9 (27/63)	1.35 (0.77-2.35)
Area deprivation index^b^				
Q1	19.4 (98/505)	1.0 [Reference]	40.4 (55/136)	1.0 [Reference]
Q2	18.2 (225/1238)	0.92 (0.70-1.21)	36.8 (132/359)	0.86 (0.56-1.31)
Q3	17.5 (125/714)	0.88 (0.65-1.20)	34.0 (86/253)	0.76 (0.48-1.19)
Q	11.7 (14/120)	0.55 (0.28-1.01)	31.7 (13/41)	0.68 (0.30-1.51)

Abbreviations: OR, odds ratio; Q, quartile (Q4 represents highest area deprivation).

^a^ Includes American Indian and Alaskan Native, Native Hawaiian and Pacific Islander, Other Pacific Islander, Samoan, unable to provide, unknown, chose not to disclose, and other unspecified.

^b^ Cochran-Armitage tests for trend are significant for vaccine initiation and completion.

### Vaccine Initiation

**Figure 1.**
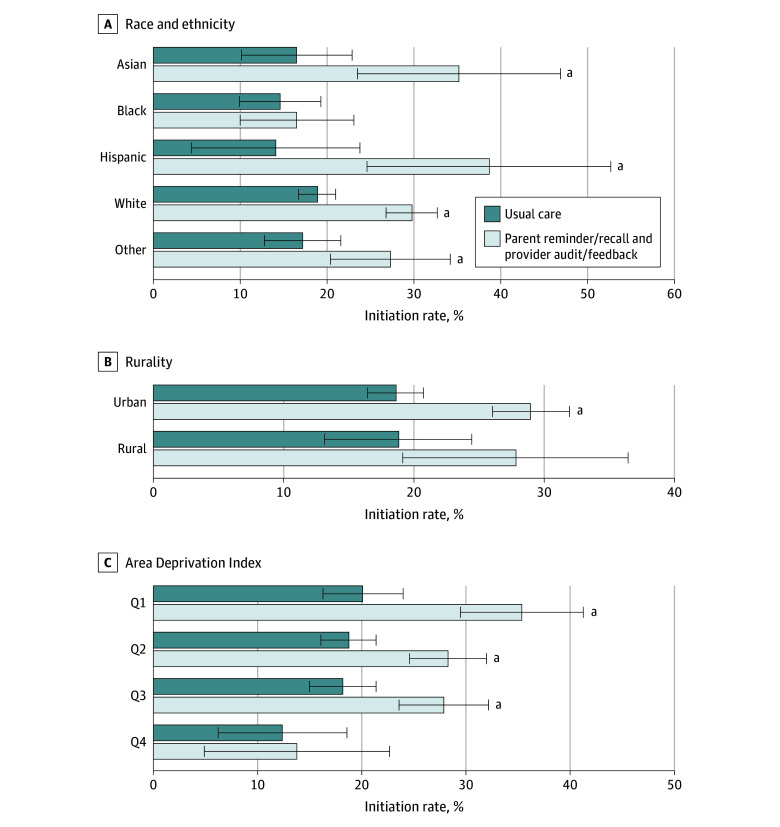
Human Papillomavirus Vaccine Initiation Rates by Characteristics of 4443 Study Participants Initiating Vaccines Error bars indicate 95% CIs. Q indicates quartile (Q4 represents highest area deprivation). ^a^A significant (*P* < .05) difference in odds of human papillomavirus vaccine initiation with reminder/recall and audit/feedback compared with usual care.

Compared with usual care, vaccine initiation rates increased with implementation of reminder/recall and audit/feedback (Figure 1). With the intervention, vaccine initiation increased significantly for most children (range of rates, 9.2% [95% CI, 5.2%-13.3%] to 24.0% [95% CI, 7.5%-40.6%]) except those with Black race, in rural settings, and in ADI Q4 (highest area deprivation) (Figure 1; eTable 2 in Supplement 1). The intracluster correlation ranged from 0.0 to 0.018.

### Vaccine Completion

**Figure 2.**
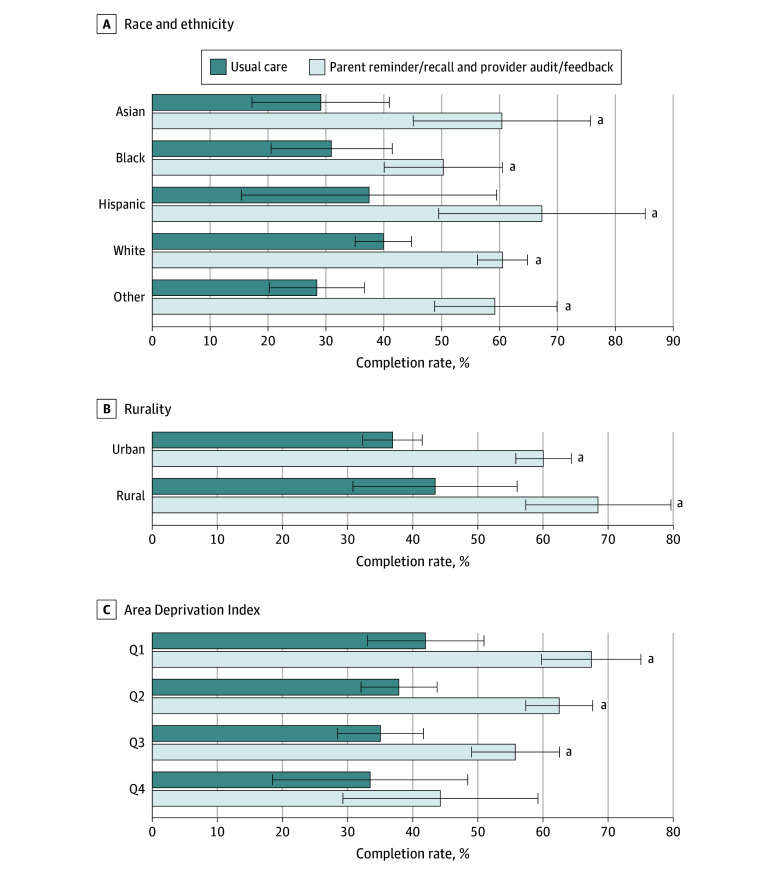
Human Papillomavirus Vaccine Completion Rates by Characteristics of 1789 Study Participants Completing Vaccines Error bars indicate 95% CIs. Q indicates quartile (Q4 represents highest area deprivation). ^a^A significant (*P* < .05) difference in odds of HPV vaccine completion with reminder/recall and audit/feedback compared with usual care.

Compared with usual care, HPV vaccine completion rates also increased with the interventions. When stratified by race and ethnicity, rurality, and ADI, vaccine completion rates increased significantly among all children (range of rates, 19.4% [95% CI, 5.5%-33.3%] to 31.2% [95% CI, 12.1%-50.3%]) except for those who were living in the most deprived areas (Figure 2; eTable 3 in Supplement 1). The intracluster correlation ranged from 0.0 to 0.017.

### Heterogeneity of Effect

**Figure 3.**
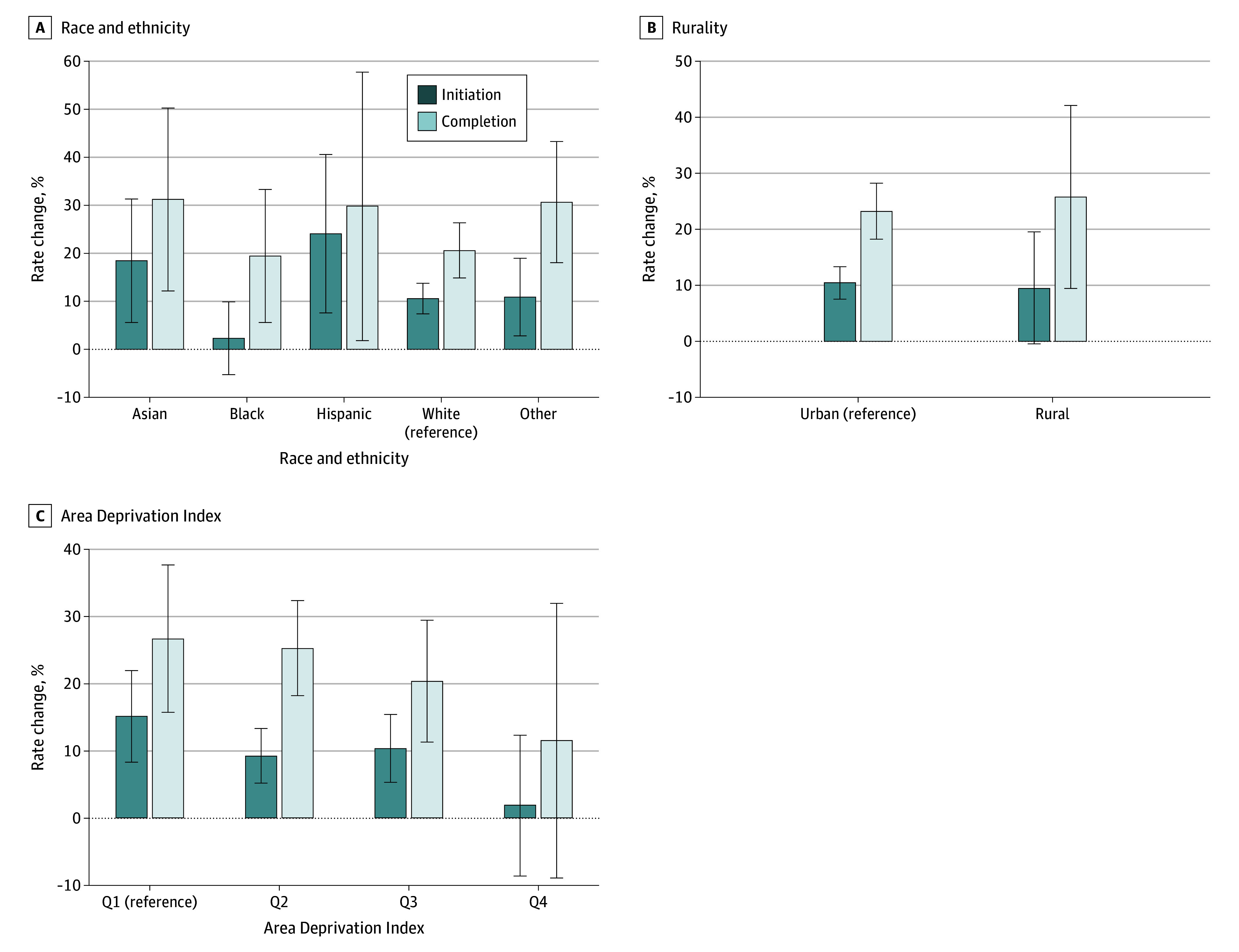
Change in Human Papillomavirus Vaccination Rates With Interventions vs Usual Care by Characteristics of All 6232 Study Participants Error bars indicate 95% CIs. Q indicates quartile (Q4 represents highest area deprivation).

Figure 3 shows changes in initiation and completion rates by adolescent characteristics. Initiation rates increased in all groups after the interventions, with the least improvement for Black children (377 [2.2%]) and the most for Hispanic children (100 [24.0%]). Completion rate improvements were lowest for Black children (184 [19.4%]) and highest for Asian children (99 [31.2%]). Initiation and completion rates improved similarly among urban and rural children. Improvements in initiation and completion rates were highest among children living in the least deprived areas (ADI Q1) and lowest in the most deprived areas (Q4). However, none of the interaction terms were statistically significant.

## Discussion

We sought to determine whether the effect of parent reminder/recall and health care professional audit/feedback on HPV vaccination varied by adolescent characteristics. Similar to a previous observational study,^8^ we found an association between area deprivation and HPV vaccination with usual care wherein lower vaccination rates were observed in areas of higher deprivation. Consistent with recommendations from the Community Preventive Services Task Force on implementing evidence-based strategies in combination,^35^ our multilevel intervention improved HPV vaccine initiation and completion among children residing in ADI Q1 to Q3. However, these improvements did not differ significantly across ADI Qs. Furthermore, our interventions did not significantly improve initiation or completion rates in the most deprived areas. Our findings underscore the importance of addressing place-based variations in HPV vaccination and the need for research to specifically identify barriers faced by patients and health care professionals in the most deprived areas.

We observed consistent decreases in vaccine initiation and completion, with the lowest rates among children in the most deprived areas—both with usual care and after the interventions—compared with those in the least deprived areas. Although the findings were nonsignificant, we noted that the improvements in vaccination were uneven across ADI Qs. With few studies assessing HPV vaccination by area deprivation,^8,9,10,12^ these findings highlight an opportunity to explore the unique individual, social, and structural barriers to HPV vaccination across different area deprivation levels. Potential mechanisms between ADI and vaccination to explore in subsequent studies may include socioeconomic barriers, normative beliefs, time availability for clinic visits, and proximity to practices. Future studies should evaluate the role of alternative settings, such as community pharmacies,^36^ convenient care clinics,^37^ and school-based health centers,^38^ in bolstering efforts to increase vaccination. In addition, raising parents’ awareness of their child’s due HPV vaccination and health care professionals’ awareness of their HPV vaccine performance was not sufficient to increase vaccination in ADI Q4, which had the lowest rates with usual care. The nonsignificant effects could be due to the small number (4.2%) of eligible children in Q4. Additional studies are needed to identify appropriate strategies for children in the most deprived areas who could benefit greatly from effective HPV vaccine promotion efforts. Formative research with parents, particularly those in the highest deprivation areas, are also needed to assess how interventions could be tailored to address facilitators and barriers to vaccination among this community.

Although the nonsignificant differences by adolescent race and ethnicity as well as rurality observed with usual care were inconsistent with previous findings,^7,8,9,10,11,12^ the increased HPV vaccine uptake across these characteristics indicate that our interventions were effective in promoting vaccine initiation and completion for most children. However, improvements in HPV vaccination were lowest for Black children. Although these changes are qualitatively different from those among White children, the interaction terms were not statistically significant. The nonsignificant interactions suggest that the intervention impact did not differ by race and ethnicity, pointing to the limitation of our interventions that were designed to increase HPV vaccination universally across children. On the other hand, these findings suggest that the interventions did not inadvertently promote HPV vaccination disparities. Another potential explanation for the nonsignificant interactions is that we may not have had the power to detect statistically significant differences, given the smaller number (9.0%) of Black children compared with the much larger sample (72.2%) of White children. Larger samples of children by race and ethnicity are needed in future studies to assess the intervention effects across race, ethnicity, and intersecting identities. If validated in the future, such results could highlight the need for additional studies to identify and tailor intervention strategies for different adolescent subgroups to increase HPV vaccination more equitably across all age-eligible children.

Notably, improvements in HPV vaccine completion were consistently higher than in vaccine initiation across all subgroups. Although our interventions targeted factors of vaccination, such as strong recommendations and fear of needles, identified by previous work and formative research with parents,^23^ findings from this analysis suggest that the interventions were more effective in promoting vaccine completion than vaccine initiation. This difference in intervention effectiveness could be attributed to our reminder/recall letter not addressing other correlates, such as parental beliefs about HPV vaccination that are associated distinctly with vaccine initiation in other work,^39^ which may have preexisted as possible barriers before health care professionals had the opportunity to address at a well visit when the child first became vaccine eligible. Thus, when conducting formative research to tailor intervention strategies, interventionists could consider developing different approaches targeted at encouraging vaccine initiation vs completion.

### Limitations

Our analysis adds to the extant intervention literature by assessing how parent reminder/recall and health care professional audit/feedback impact HPV vaccine initiation and completion across different adolescent populations. However, we note some key limitations of this study. First, this study is a secondary analysis of a trial conducted among 6 primary care clinics that served children in largely urban settings. This post hoc analysis was not part of the original trial design; therefore, we lacked statistical power to detect differences among smaller strata. Our findings suggest that future studies should include larger samples of children in rural and most deprived areas to understand their barriers to HPV vaccine initiation and completion. Second, because our analysis included all patients who were empaneled at the time of their 11th or 12th birthday, those who relocated during the trial period were determined as not having a positive vaccination outcome, which may have resulted in artificially lower vaccination rates in our study. However, this potential bias is unlikely to differ across the trial arms. Third, clinical practices for HPV vaccination and patient demographics at the 6 participating clinics may be different from other health systems and practices; hence, the generalizability of our findings may be limited. An evaluation of reminder/recall and audit/feedback in other health care settings can further inform the utility of these interventions. Fourth, the interventions were implemented at the practice level to reduce risk of contamination within practices, but the psychosocial characteristics of patients and health care professionals may differ across practices and potentially modify the effect of the interventions. Fifth, vaccination rates among participant subgroups in our study may have been affected differently by potential vaccine hesitancy resulting from the COVID-19 pandemic. Additionally, we examined patient characteristics that were available in the EHRs and hence omitted other potentially relevant characteristics in the analysis. Future studies could consider individual-level socioeconomic status that is associated with HPV vaccine initiation and completion^40^ to provide additional insights for future interventions.

## Conclusions

Parent reminder/recall letters and health care professional audit/feedback reports are effective for improving HPV vaccination among most 11- to 12-year-olds. However, the low vaccine initiation and completion rates among children in areas of highest deprivation underscore the importance of identifying barriers to HPV vaccination in this population. Such data can be used to guide development of effective interventions to promote equitable vaccine coverage.

## Notes

Supplement 1. **eFigure.** The Consolidated Standards of Reporting Trials Flow Diagram **eTable 1.** Timeline of the Stepped-Wedge Cluster Randomized Trial **eTable 2.** Odds of HPV Vaccine Initiation by Participant Characteristics (n = 4,443) **eTable 3.** Odds of HPV Vaccine Completion by Participant Characteristics (n = 1,789)

Supplement 2.
Trial Protocol

Supplement 3.
Data Sharing Statement
