# Supplementary material for: Multilevel Intervention and Human Papillomavirus Vaccination Disparities: A Secondary Analysis of a Cluster Randomized Trial
Source: JAMA Netw Open. 2025 Jul 7;8(7):e2518895. doi: 10.1001/jamanetworkopen.2025.18895 (PMC12235494; doi:10.1001/jamanetworkopen.2025.18895)
Supplement: Supplement 1. — eFigure. The Consolidated Standards of Reporting Trials Flow Diagram eTable 1. Timeline of the Stepped-Wedge Cluster Randomized Trial eTable 2. Odds of HPV Vaccine Initiation by Participant Characteristics (n = 4,443) eTable 3. Odds of HPV Vaccine Completion by Participant Characteristics (n = 1,789) [file jamanetwopen-e2518895-s001.pdf]

Supplemental Online Content

Kong WY, Finney Rutten LJ, Herrin J, et al. Effectiveness of a multilevel intervention on human papillomavirus vaccination disparities: a secondary analysis of a cluster randomized trial. *JAMA Netw Open*. 2025;8(7):e2518895.
doi:10.1001/jamanetworkopen.2025.18895

eFigure. The Consolidated Standards of Reporting Trials Flow Diagram

eTable 1. Timeline of the Stepped-Wedge Cluster Randomized Trial

eTable 2. Odds of HPV Vaccine Initiation by Participant Characteristics (*n*= 4,443)

eTable 3. Odds of HPV Vaccine Completion by Participant Characteristics (*n*= 1,789)

This supplemental material has been provided by the authors to give readers additional information about their work.

© 2025 Kong WY et al. *JAMA Network Open.*

Supplemental eFigure 1. The Consolidated Standards of Reporting Trials flow diagram

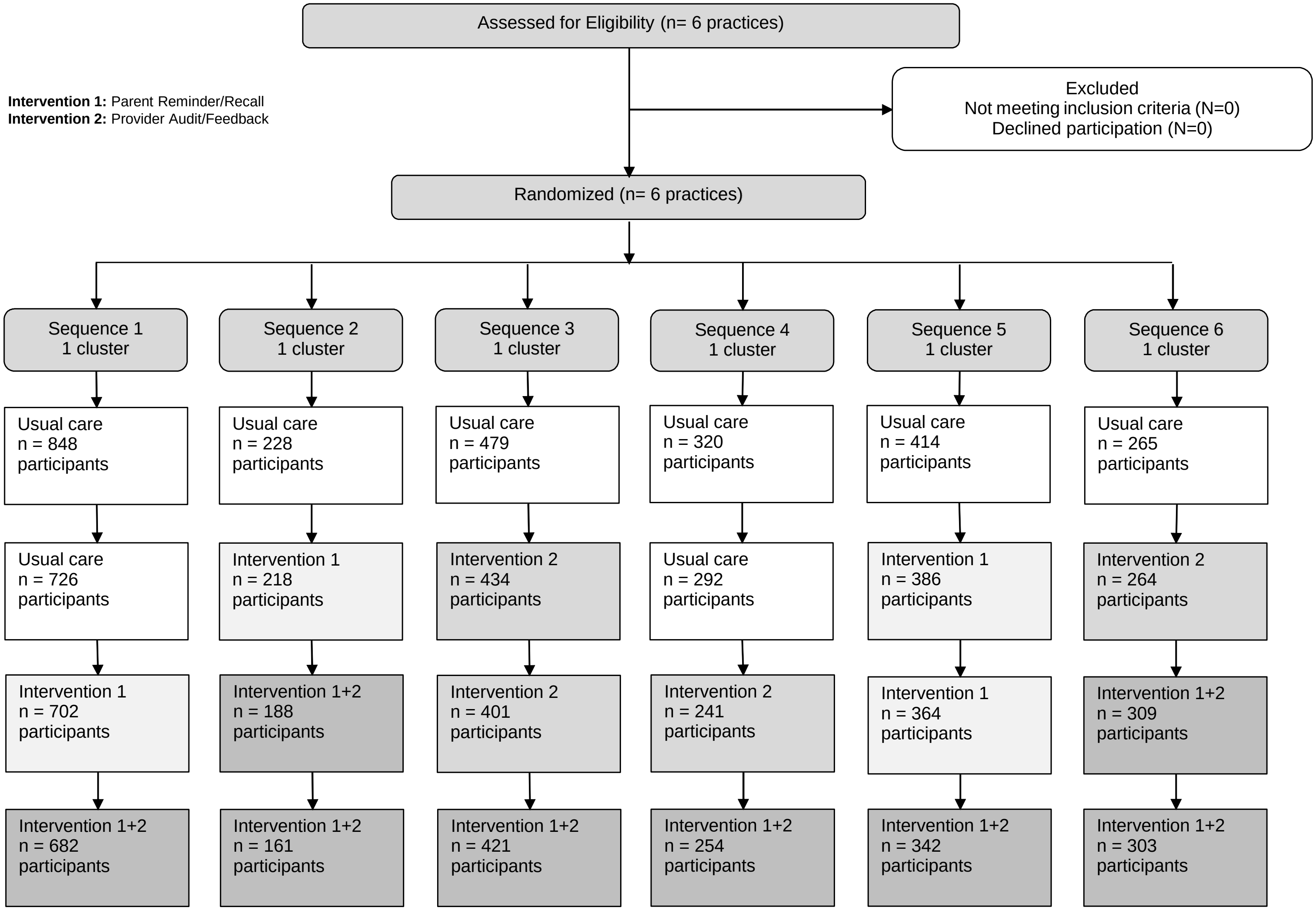

© 2025 Kong WY et al. *JAMA Network Open.*

Supplemental eTable 1. Timeline of the stepped-wedge cluster randomized trial

Practice	Step 1: April 1, 2018-March 31, 2019	Step 2: April 1, 2019-March 31, 2020	Step 3: September 1, 2020-August 30, 2021	Step 4: September 1, 2021-August 31, 2022
1	Usual care	Usual care	Parent reminder-recall	Parent reminder-recall and provider audit-feedback
2	Usual care	Parent reminder-recall	Parent reminder-recall and provider audit-feedback	Parent reminder-recall and provider audit-feedback
3	Usual care	Provider audit-feedback	Provider audit-feedback	Parent reminder-recall and provider audit-feedback
4	Usual care	Usual care	Provider audit-feedback	Parent reminder-recall and provider audit-feedback
5	Usual care	Parent reminder-recall	Parent reminder-recall	Parent reminder-recall and provider audit-feedback
6	Usual care	Provider audit-feedback	Parent reminder-recall and provider audit-feedback	Parent reminder-recall and provider audit-feedback

© 2025 Kong WY et al. *JAMA Network Open.*

Supplemental eTable 2. Odds of HPV vaccine initiation by participant characteristics (*n*=4,443)

Characteristic	Usual care	Parent reminder-recall and provider audit-feedback OR (95% CI)	*p*-value	Intracluster correlation
Race/ethnicity				
Asian	Reference	**2.76 (1.40, 5.44)**	0.003	0.000
Black	Reference	1.19 (0.66, 2.15)	0.56	0.000
Hispanic	Reference	**3.86 (1.44, 10.36)**	0.007	0.000
White	Reference	**1.78 (1.50, 2.12)**	<0.001	0.004
Other[a]	Reference	**1.91 (1.20, 3.05)**	0.007	0.018
Rurality				
Urban	Reference	**1.79 (1.53, 2.10)**	<0.001	0.003
Rural	Reference	1.72 (0.99, 3.00)	0.06	0.000
Area deprivation index				
Q1	Reference	**2.16 (1.54, 3.02)**	<0.001	0.017
Q2	Reference	**1.68 (1.34, 2.09)**	<0.001	0.006
Q3	Reference	**1.82 (1.37, 2.42)**	<0.001	0.011
Q4	Reference	1.19 (0.47, 3.01)	0.72	0.000

Note. HPV = human papillomavirus virus; OR = odds ratio; Q = quartile (Q4 represents highest area deprivation).
Boldface indicates *p*-value < 0.05.
[a]Includes American Indian and Alaskan Native, Native Hawaiian and Pacific Islander, Other Pacific Islander, Samoan, unable to provide, unknown, chose not to disclose, and other unspecified.

© 2025 Kong WY et al. *JAMA Network Open.*

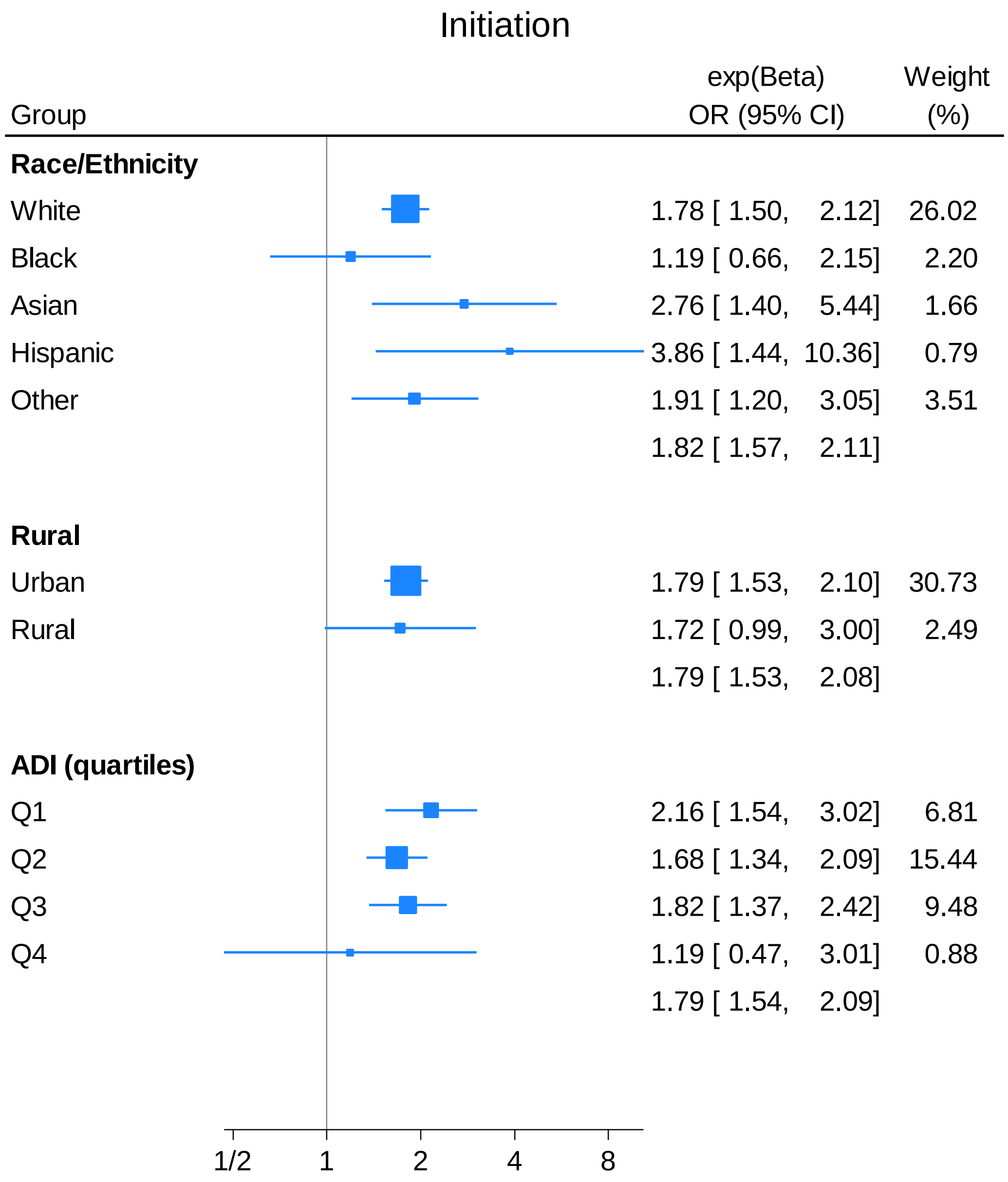

© 2025 Kong WY et al. *JAMA Network Open.*

Supplemental eTable 3. Odds of HPV vaccine completion by participant characteristics (*n*=1,789)

**Characteristic**	**Usual care**	**Parent reminder-recall and provider audit-feedback OR (95% CI)**	***p*-value**	**Intracluster correlation**
Race/ethnicity				
Asian	Reference	**3.71 (1.59, 8.64)**	0.002	0.000
Black	Reference	**2.27 (1.24, 4.19)**	0.008	0.000
Hispanic	Reference	**3.42 (1.00, 11.70)**	0.05	0.000
White	Reference	**2.30 (1.81, 2.91)**	<0.001	0.003
Other[a]	Reference	**3.66 (2.08, 6.42)**	<0.001	0.000
Rurality				
Urban	Reference	**2.57 (2.08, 3.19)**	<0.001	0.007
Rural	Reference	**2.91 (1.43, 5.92)**	0.003	0.000
Area deprivation index				
Q1	Reference	**3.00 (1.87, 4.82)**	<0.001	0.000
Q2	Reference	**2.81 (2.08, 3.79)**	<0.001	0.017
Q3	Reference	**2.30 (1.57, 3.37)**	<0.001	0.007
Q4	Reference	1.64 (0.67, 3.98)	0.28	0.000

Note. HPV = human papillomavirus virus; OR = odds ratio; Q = quartile (Q4 represents highest area deprivation).
Boldface indicates *p*-value < 0.05.
[a]Includes American Indian and Alaskan Native, Native Hawaiian and Pacific Islander, Other Pacific Islander, Samoan, unable to provide, unknown, chose not to disclose, and other unspecified.

© 2025 Kong WY et al. *JAMA Network Open.*

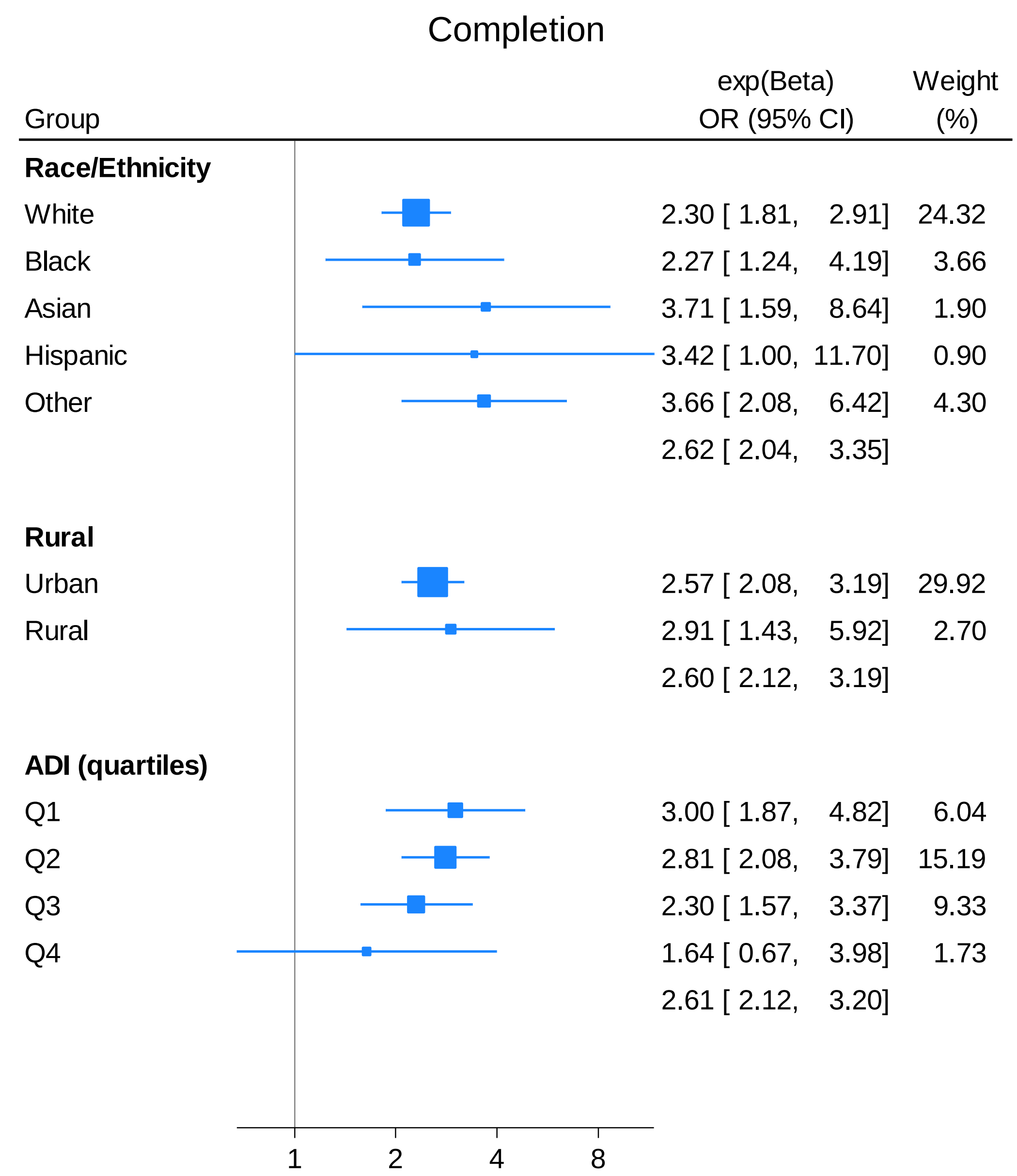

© 2025 Kong WY et al. *JAMA Network Open.*
