# Supplementary material for: Multilevel Intervention and Human Papillomavirus Vaccination Disparities: A Secondary Analysis of a Cluster Randomized Trial
Source: JAMA Netw Open. 2025 Jul 7;8(7):e2518895. doi: 10.1001/jamanetworkopen.2025.18895 (PMC12235494; doi:10.1001/jamanetworkopen.2025.18895)
Supplement: Supplement 2. — Trial Protocol [file jamanetwopen-e2518895-s002.pdf]

IRB Minimal Risk Protocol Template

General Study Information

Co-Principal Investigators: Robert M Jacobson, MD and Lila J Finney Rutten, PhD

Study Title: Multilevel Interventions for Patient, Parent, and Practice to Enhance Provider Recommendations for HPV Vaccination – Phase 3: Impact of "Less Pain, Less Fuss, Right Now!" and "Make It Count!"

Protocol version number <u>and date</u>: version 0.8, May 2, 2024

Research Question and Aims

Hypothesis: Our overarching hypothesis is that multilevel interventions for patient, parent, and practice can enhance provider recommendations for HPV vaccination.

Aims, purpose, or objectives: We are testing this hypothesis through three phases for which we have developed three protocols. In this third phase, with this protocol, we have three primary aims.

Aim 1: Test the hypothesis that, as compared to no intervention, a practice-level intervention utilizing reminder-recalls featuring the availability of non-medication and medication anesthetics, the convenience of nurse-only visits, and the use of persuasive language for early, on-time vaccinations—"Less Pain, Less Fuss, Right Now!"—will improve the odds of a child receiving an HPV vaccine dose by at least 20%.

Aim 2: Test the hypothesis that, as compared to no intervention, a provider-level intervention utilizing missed opportunities audit-and-feedback and equipping providers with a strong-recommendation toolkit—"Make It Count!"—will improve the odds of a child receiving an HPV vaccine dose by at least 20%.

Aim 3: Test the hypothesis that simultaneous implementation of interventions targeting individual, interpersonal, and organizational factors will have a synergistic effect more than doubling the odds of a child receiving an HPV vaccine dose.

Secondary aims include using process measures from the second aims from Phase 1 and Phase 2 submitted as separate IRB protocols. In these aims we will ascertain through process measures why the impacts from the interventions had the effects that they did. An additional secondary aim is to examine the impact of patient and provider characteristics on response to the study interventions.

Background: Our relevant experience includes our team's considerable work in vaccine delivery research with particular emphasis on HPV vaccination. This work has focused on parental attitudes toward HPV vaccination, population knowledge and attitudes about HPV vaccination, awareness and knowledge of HPV vaccination among uninsured, low income populations; factors associated with HPV vaccination initiation and completion, and strategies to address vaccine hesitancy. We have also studied population health interventions that health care organizations can adopt including reminder-recall strategies and others. Our research team partners closely with primary care practice leadership to identify priorities for research to improve the clinical practice and

community health. The clinical sites involved in this effort serve as a laboratory for health care delivery research, and our team has significant experience conducting research in these clinics. (See attachments 1, 2, and 3 for letters of support.)

Current knowledge suffers significant gaps. Nearly 39,000 human papillomavirus (HPV)–associated cancers occur each year including 23,000 in females and 16,000 in males. Of these, 30,700 result from HPV infection, and 28,500 result from HPV strains preventable by the highly immunogenic and efficacious 9-valent HPV vaccine. While HPV vaccination is recommended routinely for all males and females 11 to 12 years of age, HPV vaccination rates in the United States (US) are failing to reach national goals; thus, leaving millions at risk for HPV-associated cancer. Rates of HPV vaccine series completion are significantly lower than rates observed for the other two adolescent vaccines introduced at approximately the same time for the same age group. The HPV vaccination rates in our community and region reflect the low rates observed in Minnesota and the rest of the US. Barriers to vaccination reported by parents of adolescents who have not started or completed the series include the beliefs that the vaccine is optional, not recommended, unnecessary, and unsafe. Because the vaccine series has involved three injections over six months in the past, the series required several visits. However, adolescents infrequently use health services; thus, they lack frequent opportunities for vaccination. Educational campaigns have failed to produce substantial improvements in HPV vaccine delivery rates. While other countries have addressed HPV vaccine delivery successfully through school-based programs, the US relies on primary-care-practice delivery of routine adolescent vaccination. Other vaccines in the US have achieved desired national goals in large part through state-based school vaccine mandates, but states have avoided mandating HPV vaccine for school attendance because of political concerns. By contrast, a number of proven practice-level interventions offer small but incremental improvements in HPV vaccine delivery rates. Reminder-recalls directed to the patients and their parents are such practice-level interventions. They have produced varying effect sizes with improving HPV vaccination rates. Tailoring the message to address both patient and parent issues with HPV vaccination offers an opportunity to improve the effect size, but this has not been tested directly. Other innovative practice-level strategies make sense in theory but have not been tested empirically—such as routinely offering medication anesthetics to make the HPV vaccination relatively painless and emphasizing nurse-only visits utilizing standing orders (or nursing protocols) to facilitate easy access.

Our preliminary data support our work. Using patient-level data geocode-matched to publically available data from the American Community Survey, we characterized HPV vaccination initiation and completion rates in our local population. We observed a greater likelihood of vaccination among females and older adolescents. We also found our composite, environmental level measure of socioeconomic status to be significantly associated with both initiation and completion, even after controlling for individual level variables known to be associated with vaccination. Across a 7-county area in southeast MN where we have sufficient population coverage to ascertain population estimates we found 4,066 (27.1%) of children and adolescents aged 9 to 14 had received one or more doses of HPV vaccine, and 1,524 (10.1%) of those of the same age had received three or more doses of HPV vaccine. Receipt of one more doses of HPV vaccine ranged from 11.7% to 33.8% across counties. Receipt of the three doses needed to complete the series ranged from 2.5% to 13.7%. These rates are similar to the rest of Minnesota and the overall US population. None of the rates approximated the 80% Healthy People 2020 goal.

Study Design and Methods

Methods:

The overall study grant of which this protocol is the third of three parts is designed as a stepped wedge cluster randomized trial. The cluster approach prevents cross-contamination between patients or between providers as we allocate two separate interventions (i.e. “Less Pain, Less Fuss, Right Now!” and “Make It Count!”) — to the six primary care practices. The stepped-wedge design permits us to test the presence of each of the interventions in each primary care practice, making trial participation more attractive to each primary care practice, while also allowing each practice to serve as its own control, reducing the bias due to imbalanced risk factors across practices. The incorporation of a factorial design allows us to use a single trial to test two interventions and assess each individually and in combination. The design also provides opportunity to conserve overall sample size while maintaining power.

Research Setting: We will conduct our study in six primary care practices (Community Pediatric and Adolescent Medicine, Family Medicine, Northeast Family Clinic, Northwest Family Clinic, Southeast Family Clinic, and Kasson Clinic) that have expressed a commitment to improving HPV vaccination rates. These practices employ salaried nurse-practitioners, pediatricians, and family physicians. Two of the practices train residents in family medicine and pediatrics. The study sites provide care to children including those in the target age group, 11 to 12 years of age. Five of the primary care practices are located in Rochester, Minnesota and one of the practices is 15.7 miles west of Rochester in Kasson, Minnesota. Uniformity across the six clinical practices will enhance the success and efficiency of the study and contribute to the fidelity of the interventions as well as the interpretability of the results. None of the practices have had or currently have campaigns in place to reduce pain of HPV vaccination, although nurses do have access to an instant topical anesthetic skin refrigerant or vapo-coolant (Gebauer's Pain Ease Mist Spray®, Gebauer Co.) that does not require a provider order and have been taught comfort holds for infants receiving vaccinations. None have a reminder-recall process in place for HPV vaccination. None have provider-performance assessments regarding HPV vaccination. All six practices ask patients receiving HPV vaccine to wait 15 minutes post vaccination to identify symptoms of syncope and utilize the same protocol for timing the 15 minutes.

Study Design: To accomplish Aims 1-3, we will use a stepped-wedge cluster randomized trial with process evaluation. The cluster approach prevents cross-contamination between patients or providers as we allocate two separate interventions to the six primary care practices. The stepped-wedge design permits us to test the presence of each of the interventions in each primary care practice, making trial participation more attractive to each primary care practice, while also allowing each practice to serve as its own control, reducing the bias due to imbalanced risk factors across practices. The incorporation of a factorial design allows us to use a single trial to test two interventions and assess each individually and in combination. The design also provides opportunity to conserve sample size while maintaining power. In addition, we will collect data from parents and providers about their experiences with the intervention components and use these process measures along with provider- and clinic-level characteristics to analyze outcomes and make adjustments necessary for future dissemination.

Allocation: We will allocate participating practices as illustrated in the table. There will be four 12-month steps in our design, for a total of 48 months. The first step will be a baseline period in which no intervention is implemented; data collected during this period will provide a within-practice control group for each practice. For the next step, two practices will be randomly selected to receive intervention 1 (Aim 1) and two practices randomly selected to receive intervention 2 (Aim 2). For the third step, the two practices with no intervention will be randomly allocated to 1 or 2; practices initially with intervention 1 will be randomly allocated to

interventions 1+2 or intervention 1; and practices initially with intervention 2 will be randomly allocated to interventions 1+2 or 2. For the final step, all practices will receive both interventions. To ensure balance of patient numbers across interventions, we will block randomize at the first step, with the six practices grouped into three pairs according to volume. Physicians, NPs, residents, and patients belong to the individual, geographical separated practices and do not practice or obtain care at more than one practice. The lack of overlap minimizes the risk of contamination.

Table: Factorial Design Utilized by the Proposed Stepped-Wedge, Cluster-Randomized Trial

Practice	Step 1	Step 2	Step 3	Step 4
A	0	0	1	1+2
B	0	1	1+2	1+2
C	0	2	2	1+2
D	0	0	2	1+2
E	0	1	1	1+2
F	0	2	1+2	1+2

0=Current care
1=”Less Pain, Less Fuss, Right Now! reminder-recall
2=”Make It Count!” missed opportunities audit-and-feedback and strong recommendation provider toolkit

Interventions: As optimized in Phase 1, the “Less Pain, Less Fuss, Right Now!” intervention consists of a program of reminder-recall communication. For practices randomized to the “Less Pain, Less Fuss, Right Now!” intervention, reminder-recall communication will go out at the beginning of each month. Each month the practice will send a secure electronic communication to the parent or legal guardian of the patient through the patient’s electronic health record portal. We will send mailed letters to parents or guardians of patients who either have opted-out of the portal messaging or who do not access the portal within one week of delivering the reminder-recall through the portal. To support the “Less Pain, Less Fuss, Right Now!” reminder-recall intervention, we will conduct a broad education of the practice staff—nurses, medical secretaries, receptionists, and clinical assistants—regarding the nature of the intervention, its goals, and its likely impact on the practice. The broad education will be conducted through supervisory communications only to practice staff in practices allocated to the intervention at the beginning of the step. As optimized in Phase 2, the “Make It Count!” intervention refers to the two components including the missed opportunities audit-and-feedback and the provision of a strong recommendation provider-toolkit, along with a broad education of the practice staff—nurses, medical secretaries, receptionists, and clinical assistants—regarding the nature of the intervention, its goals, and its likely impact on the practice. The broad education will be conducted through supervisory communications only to practice staff in practices allocated to the intervention at the beginning of the step.

Data Collection: For each 12-month time period, we will collect patient empaneled data for each participating practice in our region. Demographic and geographic data (including ethnicity, race, sex, age, insurance, street addresses and ZIP codes) will obtained electronically for all empaneled patients ages 11-12. Our administrative database will be searched electronically to identify the occurrence and dates of all HPV vaccination of children ages 11-12 in our entire system from January 1, 2006 to the end of the study using current procedural terminology (CPT) codes (90649, 90650 and 90651). Vaccinations prior to the study period will be used to identify patients who have previously completed 1, 2 or 3 HPV vaccine doses with appropriate spacing (defined according to ACIP recommendations). To identify missed opportunities, we will electronically extract data for

all visits of the 11-12 year old empaneled patients to the participating practices during the study period, including the practice visited and the provider. For the secondary analysis of process evaluations, we will use the data from Phase 1 and Phase 2 collected under their second aims to determine how the levels of fidelity drove the quantitative differences found. We will also assess how patient and provider characteristics may have impacted response to the interventions .

Resources: *Describe the available resources to conduct the research (personnel, time, facilities, mentor commitment, etc.)*: We anticipate that the NIH will fund this work from April 1, 2018 through March 31, 2023 (Grant Number: 1 R01 CA217889-01A1). As indicated in the grant itself, the facilities and resources utilized for the conduct of are within the Department of Pediatric and Adolescent Medicine, the Department of Family Medicine, the Department of Medicine, the Department of Health Sciences Research, and three enterprise-wide centers, including an National Cancer Institute (NCI)-designated Cancer Center, the Robert D and Patricia E Kern Center for the Science of Health Care Delivery (CSHCD), and the Center for Clinical and Translational Science (CCaTS). The study coordination will be through the Robert D and Patricia E Kern Center for the Science of Health Care Delivery.

☐ (1a) This is a multisite study involving Mayo Clinic and non Mayo Clinic sites. *When checked, describe in detail the research procedures or activities that will be conducted by Mayo Clinic study staff.*

☐ (1b) Mayo Clinic study staff will be engaged in research activity at a non Mayo Clinic site. *When checked, provide a detailed description of the activity that will be conducted by Mayo Clinic study staff.*

Subject Information

Target accrual: We will allocate participating practices in a stepped wedge. There will be four 12-month steps in our design, for a total of 48 months. The first step will be a baseline period in which no intervention is implemented; data collected during this period will provide a within-practice control group for each practice. For the next step, two practices will be randomly selected to receive intervention 1 (Aim 1) and two practices randomly selected to receive intervention 2 (Aim 2). For the third step, the two practices with no intervention will be randomly allocated to 1 or 2; practices initially with intervention 1 will be randomly allocated to 1+2 or 1; and practices initially with intervention 2 will be randomly allocated to 1+2 or 2. For the final step, all practices will receive both interventions. To ensure balance of patient numbers across interventions, we will block randomize at the first step, with the six practices grouped into three pairs according to volume. Physicians, NPs, residents, and patients belong to the individual, geographical separated practices and do not practice or obtain care at more than one practice. The lack of overlap minimizes the risk of contamination.

Subject population (children, adults, groups): The units of allocation as mentioned above are the six primary care practices with outcomes being measured at the individual patient-level. For each 12-month-long step, we will measure the HPV vaccination status of the eligible patients empaneled to the providers at that primary care practice.

Inclusion Criteria: 1) Empaneled in one of the six participating primary care practices 2) 11 to 12 years of age at the first day of each of the 12-month-long steps 3) Due during that 12-month-long step for at least one dose of the HPV vaccine. (For immunocompetent children (the overwhelming majority), for children less than 15 years of age, the newly revised ACIP recommendations call for two doses at zero- and six-months with the minimum interval being five months between the first and second dose. Some eligible patients may have received two doses too close together for this because of the previous three-dose recommendation was in effect until December 16, 2016, making the ACIP recommendations official. In these cases where the second dose is less than five months after the first, the individual should receive a third dose of HPV vaccine 12 weeks after the second dose and at least 24 weeks after the first dose. Thus, patients who are eligible and due comprise of three groups: those having received no valid HPV vaccine; those having received one valid HPV dose AND it has now been five calendar-months or more; and those having received two valid doses but Dose 2 was given less than five months after the first. Valid doses include doses given at nine years or older (the minimum age per ACIP) and meet the minimum intervals.

Exclusion Criteria: 1) Not empaneled in one of the six participating practices. 2) Empaneled in one of the six participating practices but less than 11 years of age or more than 12 years of age on the first day of each 12 month long step. 3) Not due during that 12-month-long step for a dose of HPV vaccine.

Waiver for Parental Permission and Child Assent: We are asking for a waiver of parental permission and child assent appealing to the exceptions permitted under 45 CFR 46 section §46.116. Given that the interventions are allocated at the practice level, the research could not practicably be carried out without the waiver, and the interventions are do not exceed minimal risk, the waiver or alteration will not adversely affect the rights and welfare of the subjects, and whenever appropriate, the participants will be provided with additional pertinent information after participation. Parental permission and child assent are impracticable with the overall sample size over the four years of study involve approximately 10,000 children 11 to 12 years of age. It would be impracticable to arrange prospective sessions that would achieve timely informational discussions and provide for documentation of parental permission and adolescent assent for all 10,000 children. Even if we could overcome the logistics, the requirement of consent would compromise scientific validity as the very act of communicating the nature of the interventions (the reminder-recall) would a) serve as an intervention itself altering subjects' behaviors and responses and b) serve to create a selection bias as it would cause parents less likely to vaccinate to opt out of the study leaving those more likely to vaccinate to opt in, biasing the findings. The use of deception is justified as any notification or invitation would serve as its own intervention and confound the impact of the communication of the reminder-recall. Parents and providers however will provide their informed consent for their participation in Phase 1 and Phase 2 including for the future use of the data from Phases 1 and 2 for the secondary analysis of process evaluation here in Phase 3.

Research Activity – Category 5

Check all that apply and complete the appropriate sections as instructed.

1. ☐ **Drug & Device**: Drugs for which an investigational new drug application is not required. Device for which (i) an investigational device exemption application is not required; or the medical device is cleared/approved for marketing and being used in accordance with its cleared/approved labeling. (Specify in the Methods section)

2. ☐ **Blood**: Collection of blood samples by finger stick, heel stick, ear stick, or venipuncture.

3. ☐ **Biological specimens other than blood**: Prospective collection of human biological specimens by noninvasive means that may include: urine, sweat, saliva, buccal scraping, oral/anal/vaginal swab, sputum, hair and nail clippings, etc.

4. ☐ **Tests & Procedures:** Collection of data through noninvasive tests and procedures routinely employed in clinical practice that may include: MRI, surface EEG, echo, ultrasound, moderate exercise, muscular strength & flexibility testing, biometrics, cognition testing, eye exam, etc. (Specify in the Methods section)

5. ☒ **Data** (medical record, images, or specimens): Research involving use of existing and/or prospectively collected data.

6. ☐ **Digital Record**: Collection of electronic data from voice, video, digital, or image recording. (Specify in the Methods section)

7. ☐ **Survey, Interview, Focus Group:** Research on individual or group characteristics or behavior, survey, interview, oral history, focus group, program evaluation, etc. (Specify in the Methods section)

☐ NIH has issued a *Certificate of Confidentiality* (COC). *When checked, provide the institution and investigator named on the COC and explain why one was requested.* ________________________

**Biospecimens** – Categories 2 and 3 -- None

(2) Collection of blood samples. When multiple groups are involved copy and paste the appropriate section below for example repeat section b when drawing blood from children and adults with cancer.

a. **From healthy, non-pregnant, adult subjects who weigh at least 110 pounds**. For a minimal risk application, the amount of blood drawn from these subjects may not exceed 550ml in an 8 week period and collection may not occur more frequently than 2 times per week.
   Volume per blood draw: _____ml
   Frequency of blood draw (e.g. single draw, time(s) per week, per year, etc.) ___________

b. **From other adults and children considering age, weight, and health of subject.** For a minimal risk application, the amount of blood drawn from these subjects may not exceed the lesser of 50 ml or 3 ml per kg in an 8 week period, and collection may not occur more frequently than 2 times per week.
   Volume per blood draw: _____ml
   Frequency of blood draw (e.g. single draw, time(s) per week, per year, etc.) ___________

(3) Prospective collection of biological specimens other than blood: ______________________________

MAYO CLINIC

Review of medical records, images, specimens – Category 5b&c

Date Range: 04/01/2017 to 03/31/2022

Check all that apply (data includes medical records, images, specimens).

☐ (5a) Only data that exists before the IRB submission date will be collected.

☒ (5b) The study involves data that exist at the time of IRB submission **and** data that will be generated after IRB submission. Include this activity in the Methods section.
Examples

- The study plans to conduct a retrospective chart review and ask subjects to complete a questionnaire.
- The study plans to include subjects previously diagnosed with a specific disease and add newly diagnosed subjects in the future.

☒ (5c) The study will use data that have been collected under another IRB protocol. Include in the Methods section and enter the IRB number from which the research material will be obtained. *When appropriate, note when subjects have provided consent for future use of their data and/or specimens as described in this protocol*.

Enter one IRB number per line, add more lines as needed

☒ Data ☐ Specimens ☐ Data & Specimens ___IRB 17-010626___

☒ Data ☐ Specimens ☐ Data & Specimens ___IRB 17-010654___

☐ Data ☐ Specimens ☐ Data & Specimens ______

☐ (5d) This study will obtain data generated from other sources. Examples may include receiving data from participating sites or an external collaborator, accessing an external database or registry, etc. Explain the source and how the data will be used in the Methods section.

☐ (6) Video audio recording: *Describe the plan to maintain subject privacy and data confidentiality, transcription, store or destroy, etc.*

HIPAA Identifiers and Protected Health Information (PHI)

Protected health information is medical data that can be linked to the subject directly or through a combination of indirect identifiers.

Recording identifiers (including a code) during the conduct of the study allows you to return to the medical record or data source to delete duplicate subjects, check a missing or questionable entry, add new data points,

etc. De-identified data is medical information that has been stripped of all HIPAA identifiers so that it cannot be linked back to the subject. De-identified data is **rarely** used in the conduct of a research study involving a chart review.

Review the list of subject identifiers below and, if applicable, check the box next to each HIPAA identifier being recorded at the time of data collection or abstraction. Identifiers apply to any subject enrolled in the study including Mayo Clinic staff, patients and their relatives and household members.

Internal refers to the subject's identifier that will be recorded at Mayo Clinic by the study staff.
External refers to the subject's identifier that will be shared outside of Mayo Clinic.

**Check all that apply:**	INTERNAL	EXTERNAL
Name	✓	
Mayo Clinic medical record or patient registration number, lab accession, specimen or radiologic image number	✓	
Subject ID, subject code or any other person-specific unique identifying number, characteristic or code that can link the subject to their medical data	✓	
Dates: All elements of dates [month, day, and year] directly related to an individual, their birth date, date of death, date of diagnosis, etc. **Note:** Recording a year only is not a unique identifier.	✓	
Social Security number		
Medical device identifiers and serial numbers		
Biometric identifiers, including finger and voice prints, full face photographic images and any comparable images		
Web Universal Resource Locators (URLs), Internet Protocol (IP) address numbers, email address		
Street address, city, county, precinct, zip code, and their equivalent geocodes	✓	
Phone or fax numbers		
Account, member, certificate or professional license numbers, health beneficiary numbers		
Vehicle identifiers and serial numbers, including license plate numbers		
**Check 'None' when none of the identifiers listed above will be recorded, maintained, or shared during the conduct of this study.** (exempt category 4)	☐ None	☒ None

We will be requesting a waiver of HIPAA and consent due to the fact that we cannot predict who will receive the vaccine and who will not and individually approaching each of the large number of patients will not be feasible.

Data Analysis

Power Statement: Stepped-wedge cluster randomization trials typically have more statistical power than other cluster randomized designs, because each cluster is able to serve as its own control, accounting directly for the within cluster correlation of outcomes. Because of the complex nature of the design, we estimate statistical

power using simulation, which accounted for potential differences in baseline rates across sites. Based on pilot data from a 12-month period, we assumed an average cluster size of 800 children (400 males and 400 females), a baseline probability of requiring any vaccination of 25% (log-odds of -1.1), and a conservative estimate of the standard deviation of the random effect of 0.5, assuming a secular treatment effect. These estimates are conservative. We have sufficient power to detect meaningful patient-centered and provider-level effects in both the sex-stratified and full cohorts. For the interaction between patient-centered and provider-level effects (with the same assumptions described above), based on the simulation, we have at least 80% power to detect an odds ratio (OR) of 1.5 or greater when looking at males and females separately and of 1.34 or greater in the full cohort. To aid in interpretation, an OR of 1.34 means that a significant interaction would be detected if the synergistic effect of the two treatments or interventions is 1.34 times higher than the product of the individual ORs. For example, with ORs of 1.28 for both interventions individually, we can detect a synergistic effect of 2.2. Although our power estimates are conservative, power is somewhat marginal (70% to detect an OR of 1.45) for the synergistic effect in the sex-stratified cohorts; however, we have sufficient power to detect an effect using the combined sample. We conducted several sensitivity analyses adjusting the terms in our model (baseline probability, random effect standard deviation and secular trend) and found small changes in our already conservative power estimates. We used average cluster instead of actual size which was reasonable given our pilot data indicate only a slight imbalance across the clusters. Our average observed total cluster size was 895, with close to 50% male and 50% female; we assumed an average cluster size of 800 (400 for each sex) to allow for decreases in eligible participants overtime as a result of the effect of our interventions.

Data Analysis Plan: We will summarize patient characteristics (including sex, age, race/ethnicity, insurance status, rurality, area deprivation level) by intervention status (baseline, 1, 2 or 1+2). All patients will be analyzed on an intention to treat status; this principle will be extended to the practice status, so that delays in implementation of an intervention will not affect the intervention status of patients. We will use generalized linear mixed models to assess the effects of the interventions. All models will be assessed both overall and stratified by sex in recognition of differential rates of HPV vaccination among males and females. Our main model will be a mixed effects logistic regression model which will allow us to test the three primary aims.

For secondary analyses, we will estimate models similar to (1) using only the initiation and completion eligible patients. For all models we will report C-statistics and between-practice variance. Because of the small number of practices, estimation of between-practice variance may be computationally difficult and/or give biased results. Thus, as a sensitivity analysis we will replicate the main model (1) for all outcomes using a generalized estimating equation (GEE); GEE models account appropriately but conservatively for clustering of outcomes, and are thus appropriate for stepped wedge cluster randomized trials. While they have less power to detect effects, they do not require making assumptions about or estimates of the between practice variance. We will incorporate into our secondary analyses measures from the process evaluations from the companion protocols (Phase 1 and Phase 2) that measure parent awareness of the reminder-recall communication, provider awareness of the missed opportunities report as well as other moderating factors to determine their roles in explaining the magnitude and direction of the results we find. We will also estimate the intervention effects stratified by other patient and provider characteristics (e.g., age, race/ethnicity, rurality, area deprivation level).

Endpoints

Primary: Our primary outcome variables are the rates of HPV-vaccine receipt for empaneled eligible males and females, measured at the end of the last day of each 12-month step. Furthermore, eligible individuals must be

due for a dose of an HPV vaccine at the start of the 12-month-long step. They could be due for the first, second, or third dose. Eligibility for a dose depends on previously received valid doses received before the beginning of the 12- month-long step. Eligibility also depends upon the amount of time that passed between the last received valid dose and the start of the 12-month-long step. Valid doses include doses given at nine years or older (the minimum age permitted by the ACIP) and meet the minimum intervals. If the individual has received no valid doses previously at age 11 or 12, then the individual is eligible for a first dose. If the individual has previously received one valid dose, and at least 5 months has passed since that dose before the start of the 12-month-long step, then the individual is eligible for a second dose. If the individual has previously received two valid doses but the second dose was given less than 5 months from the first and at least 12 weeks have passed since the second dose and 24 weeks since the first dose, both before the start of the 12-month-long step, then the individual is eligible for a third dose.

Secondary: Secondary outcome variables include the HPV vaccine-initiation and HPV vaccine-completion rates for the eligible patients empaneled to a given practice. Specifically, we will measure at the end of the last day of each 12- month-long step the initiation and completion rates for the eligible patients empaneled to a given practice who presented for care of any type to that primary care practice during the 12-month-duration of that step. To qualify for eligibility for initiation, the patient must have been 11 or 12 years of age at the beginning day of the 12-month step. In addition, they must be due for the first dose of HPV vaccine, having never received a previous dose of HPV vaccine. To qualify for eligibility for completion of the HPV vaccine series, the patient must have been 11 or 12 years of age at the beginning day of the 12-month step, and due for either the second dose 6 months after the first or due for the third dose of HPV vaccine 4 months after the second when the second was given less than five months after the first. In addition to the rates of vaccine-initiation and completion as described above, we will obtain from electronic health records rates of missed opportunities. In these rates, the denominator will include the number of encounters that took place during a given step at a particular primary care practice of those empaneled patients eligible for a dose of HPV vaccine. The numerator will includes the number of those encounters in which an HPV vaccine was not given. True contraindications for HPV vaccination are extremely rare. Thus, few encounters for patients due for an HPV vaccine should result in a missed opportunity. We will therefore not attempt to make any adjustments for contraindications in our calculation of missed opportunities. We will further categorize outcomes (vaccinations and missed opportunities) by patient characteristics (e.g., sex, age, rurality) and vaccine dose (1, 2, or 3) as well as by provider type (e.g. family physician, pediatrician). We will calculate the missed opportunities rate for those providers who are in practices randomized to receive the second intervention for Aim 2 during those steps. Missed opportunities will be summarized in brief document and will be distributed monthly via e-mail to all participating providers. The document will show the missed-opportunity number of HPV vaccine eligible patients that presented to that provider for a visit and as well as the missed opportunity rate of those encounters for which the provider did not order the HPV vaccine. The provider will also see their peers' numbers and rates of missed opportunities including the top-performing peer's rates.
