# Supplementary material for: Multilevel Intervention and Human Papillomavirus Vaccination Disparities: A Secondary Analysis of a Cluster Randomized Trial
Source: JAMA Netw Open. 2025 Jul 7;8(7):e2518895. doi: 10.1001/jamanetworkopen.2025.18895 (PMC12235494; doi:10.1001/jamanetworkopen.2025.18895)
Supplement: Supplement 3. — Data Sharing Statement [file jamanetwopen-e2518895-s003.pdf]

Data Sharing Statement

Kong. Multilevel Intervention and Human Papillomavirus Vaccination Disparities. *JAMA Netw Open*. Published July 07, 2025. doi:10.1001/jamanetworkopen.2025.18895

Data
Additional Information: The original trial was registered on ClinicalTrials.gov (NCT03501992) at https://clinicaltrials.gov/ct2/show/NCT03501992.
Data available: Yes
Data types: Deidentified participant data
How to access data: The datasets generated during this trial are not publicly available due to concerns about confidentiality and waiver of consent and assent. Data may be made available from the corresponding author upon reasonable request.
When available: With publication

Supporting Documents
Document types: Other (please specify)
Additional Information: Trial protocol and primary trial results previously published
How to access documents: Trial protocol was published in Implementation Science journal (PMCID: PMC6043954) and the primary trial results were published in JAMA Pediatrics (PMCID: PMC10957109).
When available: beginning date: 04-25-2025

Additional Information
Who can access the data: Researchers upon reasonable request
Types of analyses: Any reasonable request
Mechanisms of data availability: Deidentified data will be made available with investigator support, after approval of a proposal, with a signed data access agreement.
